# *Uncinaria stenocephala* (northern hookworm) is the major endoparasite in dogs from private dog shelters in the Balkans: presence of benzimidazole susceptible isotype-1 β-tubulin alleles

**DOI:** 10.1017/S003118202510084X

**Published:** 2025-11

**Authors:** Georgiana Deak, Betim Xhekaj, Kurtesh Sherifi, Aurora-Livia Ursache, Mariana Louro, Viorica Mircean, Jan Slapeta, Angela M. Ionică

**Affiliations:** 1 Department of Parasitology and Parasitic Diseases, Faculty of Veterinary Medicine, University of Agricultural Sciences and Veterinary Medicine of Cluj-Napoca, Cluj-Napoca, CJ, Romania; 2 Faculty of Agriculture and Veterinary, University of Prishtina, Bulevardi “Bill Clinton”, Pristina, Kosovo; 3 CIISA – Centre for Interdisciplinary Research in Animal Health, Faculty of Veterinary Medicine, University of Lisbon, LI, Avenida da Universidade Técnica, Lisbon, LI, Portugal; 4 Associate Laboratory for Animal and Veterinary Sciences (AL4AnimalS), Portugal; 5 Sydney School of Veterinary Science, Faculty of Science, The University of Sydney, New South Wales, NSW, Australia; 6 Sydney Institute for Infectious Diseases, The University of Sydney, Sydney, New South Wales, NSW, Australia; 7 Clinical Hospital of Infectious Diseases of Cluj-Napoca, Cluj-Napoca, CJ, Romania

**Keywords:** Balkan, dogs, metabarcoding, parasites, resistance, shelter, zoonosis

## Abstract

Zoonotic parasites associated with domestic dogs have been well-studied in the majority of Europe. In the Balkan region, however, there is minimal knowledge of the parasites in dogs in shelters for rehoming in other European countries. This study aimed to investigate parasitic infections in dogs from two private shelters in Pristina, Kosovo. Faecal samples were collected, representing both adult dogs (72%) and puppies (28%). Coproscopic analysis revealed that 88% of dogs were infected with at least one parasite, with hookworms being the most common. Amplicon metabarcoding targeting internal transcribed spacer (ITS)-2 rRNA gene confirmed the presence of only *Uncinaria stenocephala* in 68% of samples apparently susceptible to benzimidazoles. The canonical F167Y and Q134H isotype-1 β-tubulin of *U. stenocephala* mutations conferring benzimidazole resistance were not detected. No evidence of *Ancylostoma caninum* was detected. Molecular analysis confirmed *Giardia duodenalis* in 18% of samples, with assemblages B, D and C detected. Other parasites detected included *Cystoisospora* spp. (18%), *Toxocara canis* (4%), *Toxascaris leonina* (6%), *Trichuris vulpis* (32%), *Eucoleus aerophilus* (10%) and *Dipylidium caninum* (2%). Co-infections were identified in 48% of the samples. These findings demonstrate a high frequency of gastrointestinal parasites in shelter dogs. The presence of *U. stenocephala* and *T. vulpis* points to the challenges with monitoring and managing these parasitic infections in such settings, as these are likely translocated with the rehomed dogs. The frequency of detection of hookworms emphasizes the need for further research into the distribution of hookworms in Europe because of the emerging benzimidazole resistance on other continents.

## Graphical abstract

**Figure:**
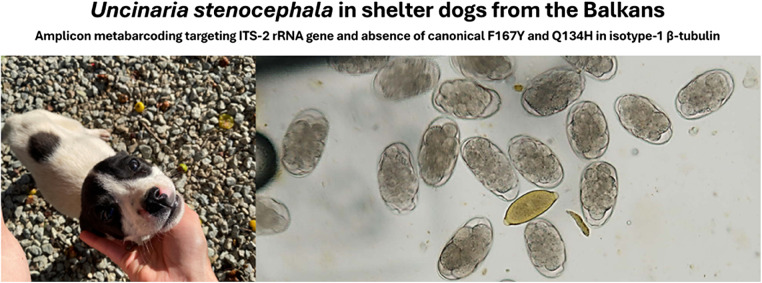

## Introduction

At a global level, about half of the population has a dog as a pet animal (Blaisdell, 1999). There is a well-known tight relationship between people and their pets, which offers benefits related to mental health, physical exercise stimulators, and socialization (Zablan et al., 2024). Zoonotic pathogens associated with domestic dogs have been well studied, especially in the current context of climate change and wildlife urbanization (Rupasinghe et al., 2022; Thompson, 2023). Many infective parasitic forms (cysts, oocysts, eggs, larvae) are dispersed with the faeces and can represent an important source of environmental contamination, considering the risk of infection for people as well (Pal and Tolawak, 2023). In the case of hookworms, in European countries, there is limited information about *Ancylostoma caninum* and *Uncinaria stenocephala* distribution across the continent (Štrkolcová et al., 2022; Illiano et al., 2023). Both *A. caninum* and *U. stenocephala* are capable of causing human infection (Pal and Tolawak, 2023). Zoonotic parasites from dogs are recognized as an important public health problem worldwide but have been mainly associated with developing countries and communities (Soriano et al., 2010).

Stray animals have an essential role as spreaders or reservoir hosts of many parasites to both animals and humans (Deplazes et al., 2011; Szwabe and Blaszkowska, 2017). In most European countries, the stray dog population was limited by implementing strategic actions like trapping-neutering-return, collect-neuter-vaccinate-return, shelters for stray dogs with limited use of euthanasia, neutering-responsible dog ownership, and microchipping (Papavasili Th et al., 2024). In other parts of Europe, such as the Balkans, stray dog management is overseen by the municipal authorities, which often do not have the proper infrastructure (Papavasili Th et al., 2024). Kosovo, especially the capital, Pristina, is known for the large population of stray dogs, which are seen as living memorials. Dogs in Kosovo are free-ranging, but the entire community supports them, and the no-killing strategy has now been adopted in the country (Ashton, 2023). The capture-neutering-vaccination-release protocol was implemented in 2017, but the country is still in economical incapacity of building specific facilities for stray dogs (Ashton, 2023). While there are currently no public dog shelters, there are several private dog shelter facilities in Pristina, which are financially supported by either organizations based in Northern Europe or individual/private donators that collect stray dogs and offer them treatment, shelter, and the chance of a better life by being adopted in higher developed European countries or USA (personal communication). Such facilities and dog management can lead to a range of health issues for dogs and are a risk of potential zoonotic transmission to humans, as they frequently house a dense population of animals with varying health statuses and unknown backgrounds (Overgaauw et al., 2020).

To investigate the extent of parasite infections in dogs destined for rehoming, the aim of this study was to investigate the proportions of parasite-positive dogs kept in private dog shelters in Prishtina, Kosovo, with a focus on hookworm diversity. Given the large population of stray dogs and their export to other countries in Europe and elsewhere, molecular tools were used to investigate the precise identity of hookworms and their susceptibility to benzimidazole by evaluating their frequency of the canonical F167Y and Q134H isotype-1 β-tubulin mutations, which confer benzimidazole resistance.

## Materials and methods

### Study group

Samples were collected from two different private dog shelters in Pristina (capital city) of Kosovo. The two shelters were randomly selected based on their availability. Shelter A hosted 32 dogs. Dogs were housed in pens placed directly on the ground. Each pen accommodated 3–6 dogs. Shelter B hosted around 300 dogs in pens with 25–35 dogs each. Each pen floor was covered with gravel. In both shelters, faeces were removed from the pens every 3 h during the day by dog keepers who were permanently present, working on shifts. Dewormings were routinely done (various unknown products) when new animals were captured and introduced into the dog shelter, and after that, only in clinically ill animals. Both shelters were euthanasia-free and promoted adoption, meaning they were housing animals of all ages and in different health conditions. In both cases, dogs were sent for adoption in European countries or USA. In instances where a dog either succumbed to a fatal disease, experienced a lethal attack by a group of dogs within an enclosure, or underwent euthanasia due to various reasons, the carcass was buried in a designated area separate from the shelter to minimize the risk of contact with other dogs. In Shelter A, which accommodated a smaller population of dogs, staff members facilitated daily walks outside the shelter. Conversely, Shelter B provided a dedicated space for walking and relaxation, an outside fenced paddock with ground and some vegetation, where dogs were taken on a rotating schedule. Following their walks, all dogs returned to their enclosures, where they remained for a 24-hour period until their next scheduled outing.

Following the current ethical and welfare regulations, faeces collection from the rectum is to be avoided; therefore, fresh faecal samples were collected from each pen as group samples/pen. A total of 50 faecal samples were analysed: 13 from Shelter A and 37 from Shelter B. Shelter A housed 32 dogs, with samples collected from 40.6% of the population. Shelter B housed approximately 300 dogs, with samples collected from 12.3% of the population.

Data about the number of animals, their sex, and approximate age of animals from each pen were noted. To avoid manipulation of the animals, and for safety reasons, age was marked according to the information received from the dog keepers and the visual inspection of animals and divided into two categories as follows: puppies (0–12 months) and adults (over 1 year).

### Parasitological diagnosis using coproscopy

Collected samples were stored in plastic ice boxes and transported to the Department of Parasitology and Parasitic Diseases from the University of Agricultural Sciences and Veterinary Medicine of Cluj-Napoca for further investigations. Each sample was processed by macroscopic observation, then individually well homogenized using a single-use wood stick. From each homogenate, 3 g were processed and microscopically analysed by Mini-FLOTAC®, using a sugar flotation solution (specific gravity [SG] = 1.27). Around 1 g of faeces from each sample was placed in 2.5 mL cryotubes and stored in a freezer at −18 for further molecular processing.

### Parasitological diagnosis using molecular tools

DNA was isolated from each sample using a commercial kit (ISOLATE II Faecal DNA kit, meridian Bioscience, London, UK), according to the manufacturer’s instructions. The amplification of various target genes of the parasites was performed by Polymerase chain reaction (PCR), using previously published primers and protocols (Table 1). The amplification set included a positive control of parasite DNA and a no-template control (PCR-grade water) to rule out possible contamination. All products were visualized by electrophoresis in 2% stained agarose gels. The size was determined by comparison to a molecular marker. DNA purification was done with a commercial kit (Gel/PCR DNA Fragments Kit, Geneaid Biotech, New Taipei, Taiwan) and externally sequenced (Macrogen Europe B.V., Amsterdam, The Netherlands). Obtained sequences were assembled and edited using Geneious software and compared to those available in the GenBank database using the Basic Local Alignment Search Tool (BLAST) analysis.

**Table 1.** Table presents all primers and protocols used in this study

Pathogen	Target gene	Product (bp)	Primers	Reference
*Giardia duodenalis*	*tpi*	605	AL3543: AAATIATGCCTGCTCGTCG	(Sulaiman et al., 2003)
			AL3546: CAAACCTTITCCGCAAACC	
		530	AL3544: CCCTTCATCGGIGGTAACTT	
			AL3545: GTGGCCACCACICCCGTGCC	
	*bg*	753	G7: AAGCCCGACGACCTCACCCGCAGTGC	(Gillhuber et al., 2013)
			G759: GAGGCCGCCCTGGATCTTCGAGACGAC	
		511	B-F: GAACGAACGAGATCGAGGTCCG	
			B-R: CTCGACGAGCTTCGTGTT	
	*gdh*	—	GDHeF: TCAACGTYAAYCGYGGYTTCCGT	
			GDHiR: GTTRTCCTTGCACATCTCC	
		432	GDHiF: CAGTACAACTCYGCTCTCGG	
			GHDiR: GTTRTCCTTGCACATCTCC	
*Dipylidium caninum*	*28S*	653	DC28S:1FGCATGCAAGTCAAAGGGTCCTACG	(Beugnet et al., 2014)
			DC28S-1R: CACATTCAACGCCCGACTCCTGTAG	
*Echinococcus multilocularis*	*nad1*	395	Cest1: TGCTGATTTGTTAAAGTTAGTGATC	(Trachsel et al., 2007)
			Cest2: CATAAATCAATGGAAACAACAACAAG	
*E. granulosus*	*rrnS*	117	Cest4: GTTTTTGTGTGTTACATTAATAAGGGTG	
			Cest5: GCGGTGTGTACMTGAGCTAAAC	
*Taenia* spp.	*rrnS*	267	Cest3: YGAYTCTTTTTAGGGGAAGGTGTG	
			Cest5: GCGGTGTGTACMTGAGCTAAAC	
Hookworms	ITS-2	331	NC-1: ACGTCTGGTTCAGGGTTGTT	(Gasser et al., 1993)
			NC-2: TTAGTTTCTTTTCCTCCGCT	
Hookworms	β-tubulin 1	293	ACB1_167_F: GGYGCAGGAAACAACTG	(Jimenez Castro et al., 2019)
			ACB1_167_R: CTTTGGTGAGGGGACAACA	
	β-tubulin 1	340	ACB1_200_F: GTRGTGGAGCCATACAATGC	(Jimenez Castro et al., 2019)
			ACB1_200_R: GGCATGAAGAAGTGAAGACGT	

### Amplicon metabarcoding and illumina deep sequencing for hookworms

All isolated DNA samples were first screened for the presence of nematode DNA using nematode ITS-2 rRNA gene quantitative PCR, and samples with Ct < 35 were considered suitable DNA material for follow-up processes. Total DNA was subjected to three independent real-time PCR amplifications. ITS-2 rRNA gene was amplified with NC-1 and NC-2 primers designed by Gasser et al. (1993) and two BZ167 and BZ200 isotype-1 β-tubulin fragments were amplified using primers designed previously (Jimenez Castro et al., 2019; Table 1). All primers were adapted for Illumina metabarcoding using next-generation sequencing (NGS). All reactions utilized SYBR-chemistry using SensiFAST™SYBR® No-ROX mix (Meridian Bioscience, Australia) and amplified as previously described by Abdullah et al. (2025), including a final melting temperature curve analysis. Each run included a no-template control (ddH_2_O) to monitor for potential contamination. Amplicon sequencing was performed at the Ramaciotti Centre for Genomics, University of New South Wales, Sydney, Australia, and sequenced on MiSeq using MiSeq Reagent Kits v2 (250PE, Illumina). De-multiplexed FastQ files were generated via BaseSpace (Illumina).

All FastQ files were processed through R package ‘dada2’ v1.34 (Callahan et al., 2016) in R v4.4.2 (R Core Team, 2022), as previously described (Abdullah et al., 2025). Before processing through the dada2 pipeline, primers were removed from all forward and reverse FastQ reads using ‘cutadapt’ version 4.0 (Martin, 2011). Resulting amplicon sequence variants (ASVs) were aggregated according to species, and samples with >2000 good quality sequence reads were considered sufficiently sequenced. The species were assigned based on ITS-2 and β-tubulin gene sequences analysed in CLC Main Workbench v22.0 (Qiagen Australia) as previously described (Stocker et al., 2024; Abdullah et al., 2025). For species assignment, an alignment of ASVs with an in-house curated reference ITS-2 rRNA gene hookworm sequence library consisting of publicly available sequences (KP844736; MT345056; JQ812692; LC036567; JX220891; EU344797; AB793527) was constructed. For β-tubulin reference sequences included *A. caninum* (DQ459314) (Schwenkenbecher et al., 2007), *A. duodenale* (EF392850), *U. stenocephala* (Stocker et al., 2023), *Necator americanus* (EU182348), and those available from ‘WormBase ParaSite’ (Howe et al., 2016; Bolt et al., 2018; Harris et al., 2019) which enabled the identity of the exon/intron boundary and amino acids Q134, F167, E198, and F200 (Venkatesan et al., 2023). New sequence data were deposited in GenBank SRA: (PRJNA1240660), and associated analysis data are available at LabArchives: https://dx.doi.org/10.25833/x7y0-fh81.

### Statistical analysis

To determine the minimum number of dogs required to detect at least one positive case with 95% confidence, the Modified Hypergeometric Exact Method was applied (Cameron and Baldock, 1998). Based on this model, a sample size of six dogs was calculated to be sufficient to detect at least one infected individual with 95% confidence, assuming a true prevalence of 40% (Epitools: https://epitools.ausvet.com.au/freecalctwo). The assumed prevalence of 40% for hookworm infections was informed by published data from dog shelters across Europe (Sommer et al., 2017; Raza et al., 2018; Štrkolcová et al., 2022; Fagundes-Moreira et al., 2025). For each shelter, the minimum detectable prevalence based on the number of samples obtained, assuming a PCR sensitivity of 95% and specificity of 100% were calculated.

The data collected was incorporated into an Excel File and the statistical analysis was done using EpiInfo™ 7.2.2.6 software (CDC, USA). The frequency, prevalence and 95% confidence interval (CI) of infection for each identified species of parasite were calculated overall and according to the shelter and age. The differences among groups were assessed by chi-square test and were statistically significant for *P* values ≤ 0.05.

## Results

### Study group

A total of 50 samples were collected across two shelters: 13 samples from Shelter A, representing 40.6% of its dog population, and 37 samples from Shelter B, accounting for 12.3% of its population. Of these, 36 samples (72%) were obtained from adult dogs and 14 (28%) from puppies across both facilities (Table 2). To contextualise the sampling effort at Shelters A and B, the probability of failing to detect the parasite if it were present at specific prevalence levels using the modified hypergeometric exact method were estimated. Assuming no positive samples were found, the probability that the parasite is present at a prevalence of ≥20% in Shelter A or ≥8% in Shelter B would be <5%, respectively.

**Table 2.** The distribution of samples collected from dogs by the shelters, age, and sex

	Shelter A		Shelter B	
	Puppy	Adult	Puppy	Adult
**M**	3	7	5	17
**F**	1	2	5	10
**Total**	4	9	10	27

### Hookworms and *Trichuris vulpis* as the most frequent parasites detected by coproscopy

Of the examined samples, 44 (88%; 95% CI 75.69–95.47%) were infected with at least one parasite. *Cystoisospora* spp. (*n* = 9, 18%), *Toxocara canis* (*n* = 2, 4%), *Toxascaris leonina* (*n* = 3, 6%), *Trichuris vulpis* (*n* = 16, 32%), hookworms (*n* = 34, 68%), *Eucoleus aerophilus* (*n* = 5, 10%) and *Dipylidium caninum* (*n* = 1, 2%) were identified using the MiniFLOTAC technique (Tables 3–5). Co-infections with at least two parasites were identified in 24 samples (48%).

**Table 3.** Table showing the overall frequencies, prevalence and 95% CI of all identified parasites

Parasite	Overall		
	Frequency	%	95% CI
‘Strongyle’ eggs	34/50	68	53.3−80.5
*Uncinaria stenocephala*	30/50	60	45.5−73.5
*Trichuris vulpis*	16/50	32	19.5−46.7
*Eucoleus aerophilus*	5/50	10	3.35−21.8
*Toxascaris leonina*	3/50	6	1.2−16.5
*Toxocara canis*	2/50	4	0.5−13.7
*Dipylidium caninum*	1/50	2	0.05−10.6
*Cystoisospora* spp.	9/50	18	8.6−31.4
*Giardia intestinalis*	9/50	18	8.6−31.4

**Table 4.** Table showing the frequencies, prevalence and 95% CI of all identified parasites based on shelter and the statistical differences between shelters

	Shelter A			Shelter B			
Parasite	Frequency	%	95% CI	Frequency	%	95% CI	*X*^2^; *P* (d.f. = 1)
‘strongyle’ type eggs	5/13	38.5	13.9−68.4	29/37	78.4	61.8−90.2	5.3; 0.01^a^
*Uncinaria stenocephala*	4/13	30.8	9.1−61.4	26/37	70.3	53−84.1	4.7; 0.02^a^
*Trichuris vulpis*	4/13	30.8	9.1−61.4	12/37	32.4	18−49.8	0; 1
*Eucoleus aerophilus*	1/13	7.7	0.2−36	4/37	10.8	3−25.4	0; 1
*Toxascaris leonina*	1/13	7.7	0.2−36	2/37	5.4	0.7−18.2	0; 1
*Toxocara canis*	0/13	0	0−24.71	2/37	5.4	0.7−18.2	0.001; 1
*Dipylidium caninum*	1/13	7.7	0.2−36	0/37	0	0−9.5	0.3; 0.3
*Cystoisospora* spp.	3/13	23	5−53.8	6/37	16.2	6.2−32	0.01; 0.7
*Giardia intestinalis*	1/13	7.7	0.2−36	8/37	21.6	9.8−38.2	0.5−0.4

a Statistically significant.

**Table 5.** Table showing the frequencies, prevalence and 95% CI of all identified parasites based on the age and the statistical differences

	Puppies			Adults			
Parasite	Frequency	%	95% CI	Frequency	%	95% CI	X2; *P* (d.f. = 1)
‘Strongyle’ type eggs	2/14	14.3	1.8−42.8	32/36	88.9	73.9−96.9	22.5; < 0.0001^a^
*Uncinaria stenocephala*	2/14	14.3	1.8−42.8	28/36	77.8	60.8−89.9	14.4; < 0.0001^a^
*Trichuris vulpis*	0/14	0	0−23.2	16/36	44.4	27.9−61.9	7.2; 0.002^a^
*Eucoleus aerophilus*	0/14	0	0−23.2	5/36	13.9	4.7−29.5	0.9; 0.3
*Toxascaris leonina*	2/14	14.3	1.8−42.8	1/36	2.8	0.07−14.5	0.8; 0.2
*Toxocara canis*	2/14	14.3	1.8−42.8	0/36	0	0−9.74	2.3; 0.07
*Dipylidium caninum*	1/14	7.1	0.2−33.9	0/36	0	0−9.74	0.2; 0.3
*Cystoisospora* spp.	7/14	50	23−76.7	2/36	5.6	0.7−18.6	10.6; 0.0009^a^
*Giardia intestinalis*	5/14	35.7	12.8−64.9	4/36	11.1	3.11−26.1	2.6; 0.09

a Statistically significant.

### Molecular analysis revealed infection with *G. Duodenalis* assemblage B

Overall, for *Giardia duodenalis*, 9 samples (18%) were confirmed by PCR amplification and sequencing with 5 (55.6%) coming from puppies and 4 (44.4%) from adult dogs. Based on the BLAST analysis, assemblage B (subassamblage B4) (syn. *G. enterica*) was identified based on the *tpi* gene in one single adult dog from Shelter B. Additionally, 3 puppies and one adult were infected with assemblage D (syn. *G. lupus*), while 2 puppies and 1 adult with assemblage C (syn. *G. canis*) with 1 single puppy dog from Shelter A infected with assemblage C.

No positive results were obtained for *D. caninum* and other cestodes (*Taenia* spp., *Echinococcus* spp.).

### *Uncinaria stenocephala* identified using ITS-2 rRNA gene amplicon metabarcoding

**Figure 1.**
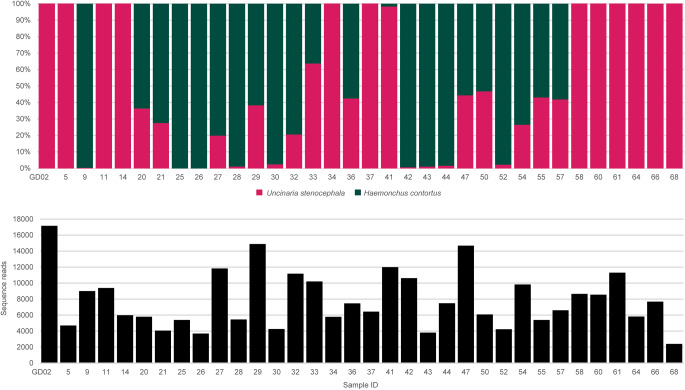
Identification of ‘strongyle’-type eggs from dogs using internal transcribed spacer (ITS)-2 rRNA gene amplicon metabarcoding assay. Percentage of nematode species within each sample (depicted as percentage). Number of sequencing reads per sample in the bar chart directly below. Sampled with hookworm-like eggs (=‘strongyle’-type eggs) were profiled using metabarcoded deep Illumina amplicon sequencing. Amplicon sequence variants (ASVs) were pooled according to species of nematodes to which they belonged using a reference sequence alignment. Proportion of each species (*Uncinaria stenocephala, Haemonchus contortus*) is colour coded in the stacked bar chart; *H. Contortus* represents spurious parasite sequences, specifically from goats/sheep.

Hookworm-like eggs (Ancylostomatidae-like, ‘strongyle’) were detected in 68% (34/50; 95% CI: 53.3–80.5%)) of the faecal samples. All 50 samples underwent DNA isolation and real-time PCR amplification of ITS-2 rRNA gene using primers targeting ‘strongyle’ DNA. The real-time PCR amplification was successful for 68% (34/50; Ct-values 19.9–30.4). Melting curve analysis revealed two dominant temperature peaks, 84°C and 81°C. There were 14 samples with a predominant or only melting peak at 84°, 10 samples with a predominant or only melting peak at 81°, and the remaining 10 samples had both peaks at 84°C and 81°C recognizable. All 34 amplicons underwent deep-sequencing using and yielded >2000 high-quality sequencing reads per samples (average: 7860, min. 2385, max. 17 148, total 257 224) (Figure 1).

The only hookworm ITS-2 rRNA gene sequences were those of *U. stenocephala* (Ancylostomatidae). There were 24 samples with >2000 sequence reads belonging to *U. stenocephala*, in addition to nine samples with <2000, and one with no detectable *U. stenocephala* ITS-2 rRNA gene reads (Figure 1). The percentage of *U. stenocephala* reads in samples ranged from 100% to 0% (average 54%) and the remaining reads were matched to *Haemonchus controtus* and considered spurious results.

The PCR amplicon with the predominant temperature melting peak of 84°C returned on average 94% reads belonging to *U. stenocephala* (min. 42%, max. 100%), and those with the predominant temperature melting peak of 81°C returned on average 5% reads belonging to *U. stenocephala* (min. 0%, max. 47%) with 9 out of 10 samples returning >97% of reads from *H. contortus*. The samples with both melting temperature peaks returned on average 40% reads belonging to *U. stenocephala* (min. 20%, max. 100%).

### *Uncinaria stenocephala* isotype-1 β-tubulin gene reveals only canonical benzimidazole susceptible residues

Further, two isotype-1 β-tubulin regions (BZ167, BZ200) that amplify region with mutations known to be associated with resistance to benzimidazole drug class, specifically the regions that encode amino acids: glutamine 134 (Q134), phenylalanine 167 (F167), glutamic acid 198 (E198), and phenylalanine 200 (F200) were amplified. Amplification was successful for 30 samples. Sequencing was successful for 16 samples (average hookworm high-quality reads 5271, min. 653, max. 18321) for BZ167 assay, and for all samples include only *U. stenocephala* sequences with canonical susceptible residues coding for Q134 and F167. Three (3/16) samples yielded <2000 high-quality hookworm reads (1447; 997; 653) at the BZ167 assay. The sequencing was successful for 12 samples (average hookworm high-quality reads 2264, min. 116, max. 6029) for BZ200 assay, and for all samples included only *U. stenocephala* sequences with canonical susceptible residues coding for E198 and F200. Three (4/12) samples yielded <2000 high-quality hookworm reads (952; 414; 318; 116) at the BZ200 assay.

## Discussion

Hookworms, ancylostomatids, were the most prevalent parasites identified by coproscopy in this study, with an overall prevalence of 68% among tested dogs. Such high prevalence is in accordance with other reports on shelter dog populations, places in which environmental contamination and close contact between animals facilitate the transmission (Mukaratirwa and Singh, 2010; Alvarado-Esquivel et al., 2015; Lopes et al., 2025). Interestingly, in studies done on European shelter dogs, hookworms were reported with a much lower prevalence: 5.3% in Spain (Ortuno and Castella, 2011) and 0.4% in Germany (Becker et al., 2012). These lower prevalences could be correlated to the proper use of prophylactic deworming and more strict environmental hygiene. However, in other geographically close countries such as Serbia and Romania, hookworms were identified with 41% and 56% prevalences in shelter dogs, respectively (Mircean et al., 2017; Sommer et al., 2017; Rusu et al., 2023), showing a lack of successful prophylactic treatments and parasite-control measures implemented.

These results revealed a high prevalence of hookworm-like eggs in dog faecal samples (68%), with *U. stenocephala* being the only hookworm species detected. Given the relatively small number of samples analysed, limiting detection to prevalence rates above 20% in Shelter A and 8% in Shelter B, it remains plausible that *Ancylostoma* spp. may be present but undetected in these shelters. This inference is supported by the use of the modified hypergeometric exact method, which estimates the probability of missing infections at lower prevalence levels. Nevertheless, the dominance of *U. stenocephala* in these findings aligns with numerous studies across Europe, which consistently report it as the predominant hookworm species in canine populations (Sommer et al., 2017; Raza et al., 2018; Štrkolcová et al., 2022; Fagundes-Moreira et al., 2025).

Interestingly, *U. stenocephala* is considered to occur in temperate/colder areas like Central and Northern Europe, Canada, and the United States, but it was also reported in tropical Africa (Bowman et al., 2010; Reinemeyer, 2016; Demkowska-Kutrzepa et al., 2018; Merino-Tejedor et al., 2019). In the United States, using the amplicon metabarcoding approach, *U. stenocephala* was shown to be a rarely detected species of hookworms regardless of the region considered, with *A. caninum* dominating the distribution of hookworms from the southern warmer climates to the cooler northern parts (Venkatesan et al., 2023; Stocker et al., 2024). The presence of *U. stenocephala* in the Mediterranean region was hypothesized to be due to the changes caused by climate change and increased animal movements (Illiano et al., 2023). In Europe, the application of molecular tools has and will significantly improve the understanding of hookworm diversity, and based on the more recent data, it is more likely to reveal that many historical records of *A. caninum* species were, in fact, *U. stenocephala*. Contrary to traditional textbook knowledge, which often presents a mix of both, molecular analyses suggest that *U. stenocephala* might be dominating across Europe or at least in the Mediterranean area, while the presence of *A. caninum* appears to be limited (Štrkolcová et al., 2022; Illiano et al., 2023). The European distribution of hookworms is unknown, while outside the European continent, distribution patterns were observed in North America (Leutenegger et al., 2024; Jimenez Castro et al., 2025) and Australia (Abdullah et al., 2025), where distinct biogeographic boundaries exist. In Australia, *U. stenocephala* is the only canine hookworm species in the South, while in the North is *A. caninum* with areas in between, like Sydney, representing the halfway meeting of hookworm species, with both species reported (Abdullah et al., 2025).

In this study, all analysed *U. stenocephala* isotype-1 β-tubulin sequences lacked the F167Y and Q134H mutations known to confer benzimidazole resistance in *A. caninum* (Venkatesan et al., 2023). There is a renewed interest in hookworms due to the multiple anthelmintic drug resistance (MADR) that has emerged (Jimenez Castro et al., 2019, 2025; Kitchen et al., 2019; Geary et al., 2025). Resistance is speculated to have emerged due to the indiscriminate use of anthelmintics, particularly in greyhound racing kennels where the first resistant *A. caninum* was detected (Von Samson-Himmelstjerna et al., 2021; Marsh and Lakritz, 2023; Geary et al., 2025). These resistant isolates are now present in other breeds as well. The widespread resistance of *A. caninum* to benzimidazole is demonstrated by phenotypic and molecular assays across North America and Australia (Venkatesan et al., 2023; Abdullah et al., 2025). Until recently, the canonical F167Y and Q134H mutations were detected only in *A. caninum*. However, the first *U. stenocephala* with the F167Y (TTC > TAC) isotype-1 β-tubulin mutation, with 51% frequency of the sequence reads, was already detected in Australia (Abdullah et al., 2025). No phenotypic evidence, whether *in vivo* faecal egg reduction tests or *in vitro* egg hatch assays, exists for benzimidazole resistance in *U. stenocephala*. Nevertheless, milbemycin oxime lacks efficacy against *U. stenocephala* (Bowman et al., 1991; Niamatali et al., 1992). Given that *U. stenocephala* is dominant in Europe and in shelters from which dogs are rehomed to other countries, the emergence of resistance under similar conditions to those where *A. caninum* MADR emerged should be considered a tangible reality. Although not widely used in Europe yet, there is a commercially available qPCR panel that can detect *A. caninum* BZ resistance (Leutenegger et al., 2023).

Dogs are known for their coprophagy. This behaviour, however, can lead to spurious parasitological results, as parasitic stages from ingested faeces can pass through the gastrointestinal tract and appear intact in the dogs’ faeces (Mircean et al., 2011). Some spurious results are generally well known by veterinary parasitology diagnosticians, such as oocysts/sporocysts of *Adelina* spp. and *Monocystis* spp. from ingested beetles and earthworms, respectively, because of their well-recognizable features (Santana-Hernández et al. 2021). For parasitic stages such as ‘strongyle’ eggs, identification can be more problematic due to the general uniformity of ‘strongyle’ eggs, regardless of their true origin (e.g. dog, rabbit and sheep). Therefore, the detection of *H. contortus* ITS-2 rRNA gene sequences in dog samples raises questions about the origin of these findings. The amplicon metabarcoding approach based on NC1-NC2 primers targeting the ITS-2 rRNA gene of nematodes (i.e. nemabiome) is capable of targeting most trichostrongylids and ancylostomatid nematodes simultaneously, thus providing an unbiased perspective on the ‘strongyle’ type eggs present in the faeces.

It is plausible that the dogs ingested *H. contortus* eggs or larvae during their time outside the shelter confinement, as both shelters were on the outskirts of the town, surrounded by pastures occupied by ruminants, *H. contortus* hosts. Additionally, the faecal sampling was done in June, when temperatures are very high and *H. contortus* contamination on pastures is at its peak. It is not, however, plausible that dogs are hosts of adult *H. contortus* because it does not infect dogs. At the PCR amplification stage, a melting peak at 81°C that correlated with the presence of *H. contortus* was noticed, suggesting that contamination post-PCR or barcode hopping during Illumina NGS is unlikely. All negative controls during the PCR remained negative, confirming that the used PCR consumables were clean and devoid of *H. contortus* DNA. Although dogs can actively ingest ruminant faeces, the faeces could have been passively contaminated because they were collected off the ground due to ethical considerations, with high larval and egg burdens from the surrounding pastures either by wind or rain. The dog keepers frequently moved between different shelter locations separated by pasture, potentially carrying parasite eggs on their shoes and introducing them into the dog enclosures. In a previous study using an analogous approach, trichostrongylid ITS-2 rRNA gene sequences were detected in several dogs and concluded to be a consequence of dogs’ coprophagy by farm dogs (Abdullah et al., 2025). In a telling analogy, cat hookworms *Ancylostoma tubaeforme* were detected in four dogs’ faeces and speculated as a consequence of dogs’ inclination to eat cat faeces (Stocker et al., 2024; Abdullah et al., 2025).

The hypothesis of coprophagy, the ingestion of faeces by dogs, is plausible but would require follow-up longitudinal evaluation. It would be expected that subsequent faeces from the same dog would have reduced or no presence of *H. contortus* while *U. stenocephala* would persist. Unfortunately, no repeated sampling was done, and the study could not fully address this hypothesis. Regardless of the underlying reason, this finding highlights the need to interpret diagnostics in parasitology carefully. The presence of ‘strongyle’-type eggs in dog faecal samples does not necessarily indicate an active hookworm infection, especially in dogs with potential access to environmental sources of contamination. Unbiased molecular survey tools, such as the ‘nemabiome’ approach, enable us to discern the presence of spurious results that traditional approaches do not provide sufficient granulation.

The identification of other prevalent nematodes, such as *T. vulpis* (32%), suggests that soil-transmitted parasites represent a significant concern in these two shelters. It is generally accepted that whipworms show some resilience to some anthelmintics and require multiple administrations and sometimes even combinations of various nematodicide molecules for an effective treatment (Traversa, 2011). It is though suggested that the evaluation of the response to treatment in shelter dogs might give a better view into potential drug resistance. Although there are some reports about *T. vulpis* infections in humans (Márquez-Navarro et al., 2012), this zoonotic disease is still considered uncommon, but should not be neglected as in heavy infections humans can develop eosinophilia, dysentery syndrome, anaemia and rectal prolapse (Márquez-Navarro et al., 2012).

*Eucoleus aerophilus* eggs were identified in only 5 dogs (10%), and no eggs of *E. boehmi* were detected. The absence of *E. boehmi* may be due to the slightly different biology of the two capillarids with *E. boehmi*, mainly localized in the nasal cavities of carnivores, making it less likely to be detected in faecal examinations. Additionally, although still controversial, *E. boehmi* has an indirect life cycle and the absence of an intermediate host in the shelter environment may further reduce the transmission (Traversa et al., 2011). Human pulmonary capilariosis due to *E. aerophilus* was occasionally reported, including in a woman misdiagnosed with bronchial carcinoma from a bordering country, Serbia (Lalosević et al., 2008).

Among ascarids, *T. leonina* (6%) was more frequent than *T. canis* (2.4%), which could be explained, by the low number of young animals examined as *T. canis* is mainly associated with young age (Baneth et al., 2016).

Although MiniFLOTAC showed good results in the detection of Metastrongylid lungworms (Ianniello et al., 2020), their transmission involves intermediate or paratenic hosts that are not commonly present in shelter environments, explaining the negative results.

*Dipylidium caninum*, the most common cestode detected in companion animals, being transmitted via fleas or lice (Beugnet et al., 2018), was identified in one single sample. The authors are aware that this finding might not reflect the actual prevalence, as the identification of such cestodes is difficult (Deak et al., 2025), as the parasitic forms are shed with the ovigerous proglottids. A recent study showed that the best technique to be used to diagnose dipylidiosis is the scotch band test from the perianal area (Morelli et al., 2024). Furthermore, negative PCR results for all tested samples, including the one positive in coproscopy, can be explained by the lack of proglottids in the tested faeces.

The absence of all *Taenia* spp. though may correlate to the lack of intermediate hosts in the shelters, as *Taenia* spp. require the consumption of rodents or other mammals for transmission, and in the present case, all dogs were fed strictly dog commercial pellets, eliminating their exposure to such parasites by the consumption of raw meat or carcasses (Deplazes et al., 2011). A second reason could be related to the DNA isolation technique, as for tapeworms eggs, it is recommended that the DNA isolation is done from egg concentrate (Trachsel et al., 2007).

A low prevalence (18%) of *G. duodenalis* was confirmed by PCR, with a higher frequency in puppies. Despite the molecular confirmation, no *Giardia* cysts were detected using the MiniFLOTAC method, which could be explained by the intermittent shedding of cysts, commonly leading to false-negative results, or the sensitivity limitations of this technique compared to the direct immunofluorescence or it was just a basic matter of cyst degradation in the faeces as samples were transported and examined two days after the collection.

In Kosovo, a limited number of studies previously investigated the parasitic fauna of dogs, and they were mainly focused on vector-borne diseases, especially *Leishmania* (Xhekaj et al., 2023), and few studies were done on *Echinococcus* spp. (Sherifi et al., 2011) in dogs.

The authors acknowledge that this study had several limitations that should be taken into consideration. First, due to the type of management system, faecal samples were collected as pooled samples from each pen rather than individually, which might have limited the correlation of parasites with each animal, in regard to the age, sex and clinical signs. Additionally, the study included two private shelters in Pristina, which might not reflect the real parasite prevalence or diversity in other regions of the country or even wider Balkans. However, the study was not designed as an epidemiological study; rather, its aim was to determine the parasitological fauna in two shelters in the vicinity of the capital that provide animals for adoption to EU countries.

## Conclusion

The present study represents the most comprehensive molecular investigations conducted in the Balkans on the parasite-positivity of shelter dogs destined for rehoming across Europe, showing that 88% of examined dogs were infected with at least one parasite, with *U. stenocephala* (northern hookworm) being the most prevalent. In the investigated samples, no benzimidazole resistance-associated mutations were found in *U. stenocephala*, indicating its susceptibility to benzimidazole drugs.

The potential indiscriminate use of benzimidazoles in shelters in Europe may mimic the conditions that led to the emergence of benzimidazole resistance in *A. caninum* in greyhound racing dog kennels (Marsh and Lakritz, 2023; Geary et al., 2025). While *U. stenocephala* resistance to benzimidazole is yet to be confirmed, mutations associated with benzimidazole resistance, mutation F167Y, has already been detected (Abdullah et al., 2025). The distribution of dog hookworms across Europe is largely assumed based on historical records that used post-mortem adult hookworms for species identification. Ante-mortem amplicon metabarcoding for hookworm identification and benzimidazole SNP screening is a suitable medium to high-throughput approach. Monitoring the susceptibility of *U. stenocephala* to benzimidazoles is crucial because milbemycin oxime is ineffective against *U. stenocephala*. Additionally, the presence of ‘strongyle’-type eggs in dogs, identified as *H. contortus* using amplicon metabarcoding, points to the importance of accurate diagnosis methods and the need to interpret the results in the clinical context and the environment the animals inhabit.

## References

[ref1] Abdullah S, Stocker T, Kang H, Scott I, Hayward D, Jaensch S, Ward MP, Jones MK, Kotze AC and Šlapeta J (2025) Widespread occurrence of benzimidazole resistance single nucleotide polymorphisms in the canine hookworm, *Ancylostoma caninum*, in Australia. *International Journal of Parasitology* 55, 173–182. 10.1016/j.ijpara.2024.12.001. 39716589

[ref2] Alvarado-Esquivel C, Romero-Salas D, Aguilar-Domínguez M, Cruz-Romero A, Ibarra-Priego N and Pérez-de-león AÁ (2015) Epidemiological assessment of intestinal parasitic infections in dogs at animal shelter in Veracruz, Mexico. *Asian Pacific Journal of Tropical Biomedicine* 5, 34–39. 10.1016/S2221-1691(15)30167-2.

[ref3] Ashton JC (2023) Following the dogs of Prishtina: Landscape as living memorial. *Landscape Research* 48, 677–690. 10.1080/01426397.2022.2150158.

[ref4] Baneth G, Thamsborg SM, Otranto D, Guillot J, Blaga R, Deplazes P and Solano Gallego L (2016) Major parasitic zoonoses associated with dogs and cats in Europe. *Journal of Comparative Pathology* 155, S54–S74. 10.1016/j.jcpa.2015.10.179. 26687277

[ref5] Becker AC, Rohen M, Epe C and Schnieder T (2012) Prevalence of endoparasites in stray and fostered dogs and cats in Northern Germany. *Parasitology Research* 111, 849–857. 10.1007/s00436-012-2909-7. 22526289

[ref66] Beugnet F, Labuschagne M, Fourie J, Jacques G, Farkas R, Cozma V, Halos L, Hellmann K, Knaus M and Rehbein S (2014) Occurrence of Dipylidium caninum in fleas from client-owned cats and dogs in Europe using a new PCR detection assay. *Veterinary Parasitology* 205(1-2), 300–306. 10.1016/j.vetpar.2014.06.008 24986432

[ref6] Beugnet F, Labuschagne M, Vos CD, Crafford D and Fourie J (2018) Analysis of *Dipylidium caninum* tapeworms from dogs and cats, or their respective fleas: Part 2. Distinct canine and feline host association with two different *Dipylidium caninum* genotypes. *Parasite* 25, 31. 10.1051/parasite/2018029. 29806593 PMC6013090

[ref7] Blaisdell JD (1999) The Rise of Man’s best friend: The popularity of dogs as companion animals in late eighteenth-century London as reflected by the dog tax of 1796. *Anthrozoös* 12, 76–87. 10.2752/089279399787000363.

[ref8] Bolt BJ, Rodgers FH, Shafie M, Kersey PJ, Berriman M and Howe KL (2018) Using WormBase ParaSite: An integrated platform for exploring helminth genomic data. In Kollmar M (ed.), *Eukaryotic Genomic Databases, Methods in Molecular Biology*. New York, New York, NY: Springer, pp. 471–491. 10.1007/978-1-4939-7737-6_15 29761467

[ref9] Bowman DD, Lin DS, Johnson RC and Hepler DI (1991) Effects of milbemycin oxime on adult *Ancylostoma caninum* and *Uncinaria stenocephala* in dogs with experimentally induced infections. *American Journal of Veterinary Research* 52, 64–67. 10.2460/ajvr.1991.52.01.64. 2021256

[ref10] Bowman DD, Montgomery SP, Zajac AM, Eberhard ML and Kazacos KR (2010) Hookworms of dogs and cats as agents of cutaneous larva migrans. *Trends in Parasitology* 26, 162–167. 10.1016/j.pt.2010.01.005. 20189454

[ref11] Callahan BJ, McMurdie PJ, Rosen MJ, Han AW, Johnson AJA and Holmes SP (2016) DADA2: High-resolution sample inference from Illumina amplicon data. *Nature Methods* 13, 581–583. 10.1038/nmeth.3869. 27214047 PMC4927377

[ref12] Cameron AR and Baldock FC (1998) A new probability formula for surveys to substantiate freedom from disease. *Preventive Veterinary Medicine* 34, 1–17. 10.1016/S0167-5877(97)00081-0. 9541947

[ref13] Deak G, Györke A, Pop CD and Mircean V (2025) Improving cestode diagnosis in domestic dogs and cats: The need for accurate and non-invasive techniques. *Preventive Veterinary Medicine* 244, 106654. 10.1016/j.prevetmed.2025.106654. 40812035

[ref14] Demkowska-Kutrzepa M, Szczepaniak K, Dudko P, Roczeń-Karczmarz M, Studzińska M, Żyła S and Tomczuk K (2018) Determining the occurrence of the *Uncinaria stenocephala* and *Ancylostoma caninum* nematode invasion in dogs in Poland, with special emphasis on the Lublin region. *Medycyna Weterynaryjna* 74, 526–531. 10.21521/mw.6104.

[ref15] Deplazes P, Van Knapen F, Schweiger A and Overgaauw PAM (2011) Role of pet dogs and cats in the transmission of helminthic zoonoses in Europe, with a focus on echinococcosis and toxocarosis. *Veterinary Parasitology* 182, 41–53. 10.1016/j.vetpar.2011.07.014. 21813243

[ref16] Fagundes-Moreira R, Bezerra-Santos MA, Alves MH, Palazzo N, Lia RP, Mendoza-Roldan JA, Šlapeta J and Otranto D (2025) *Ancylostoma caninum* and *Uncinaria stenocephala* hookworms: Morphological and molecular differentiation and epidemiological data in Southern Italy. *Veterinary Parasitology* 339, 110581. 10.1016/j.vetpar.2025.110581. 40829491

[ref17] Gasser RB, Chilton NB, Hoste H and Beveridge I (1993) Rapid sequencing of rDNA from single worms and eggs of parasitic helminths. *Nucleic Acids Research* 21, 2525–2526. 10.1093/nar/21.10.2525. 8506152 PMC309567

[ref18] Geary TG, Drake J, Gilleard JS, Chelladurai JRJJ, Castro PDJ, Kaplan RM, Marsh AE, Reinemeyer CR and Verocai GG (2025) Multiple anthelmintic drug resistance in the canine hookworm *Ancylostoma caninum*: AAVP position paper and research needs. *Veterinary Parasitology* 338, 110536. 10.1016/j.vetpar.2025.110536. 40596793

[ref64] Gillhuber J, Pallant L, Ash A, Thompson R Andrew, Pfister K and Scheuerle M C (2013) Molecular identification of zoonotic and livestock-specific Giardia-species in faecal samples of calves in Southern Germany. *Parasites Vectors* 6(1). 10.1186/1756-3305-6-346 PMC4029387 24326081

[ref19] Harris TW, Arnaboldi V, Cain S, Chan J, Chen WJ, Cho J, Davis P, Gao S, Grove CA, Kishore R, Lee RYN, Muller HM, Nakamura C, Nuin P, Paulini M, Raciti D, Rodgers FH, Russell M, Schindelman G, Auken KV, Wang Q, Williams G, Wright AJ, Yook K, Howe KL, Schedl T, Stein L and Sternberg PW (2019) WormBase: A modern model organism information resource. *Nucleic Acids Research* 48(D1), D762–D767. 10.1093/nar/gkz920. PMC7145598 31642470

[ref20] Howe KL, Bolt BJ, Cain S, Chan J, Chen WJ, Davis P, Done J, Down T, Gao S, Grove C, Harris TW, Kishore R, Lee R, Lomax J, Li Y, Muller HM, Nakamura C, Nuin P, Paulini M, Raciti D, Schindelman G, Stanley E, Tuli MA, Van Auken K, Wang D, Wang X, Williams G, Wright A, Yook K, Berriman M, Kersey P, Schedl T, Stein L and Sternberg PW (2016) WormBase 2016: Expanding to enable helminth genomic research. *Nucleic Acids Research* 44, D774–D780. 10.1093/nar/gkv1217. 26578572 PMC4702863

[ref21] Ianniello D, Pepe P, Alves LC, Ciuca L, Maurelli MP, Amadesi A, Bosco A, Musella V, Cringoli G and Rinaldi L (2020) Why use the mini-FLOTAC to detect metastrongyloid larvae in dogs and cats? *Acta Parasitologica* 65, 546–549. 10.2478/s11686-020-00171-9. 31970621

[ref22] Illiano S, Ciuca L, Maurelli MP, Pepe P, Caruso V, Bosco A, Pennacchio S, Amato R, Pompameo M and Rinaldi L (2023) Epidemiological and molecular updates on hookworm species in dogs from southern Italy. *BMC Veterinary Research* 19, 204. 10.1186/s12917-023-03765-3. 37833701 PMC10571300

[ref23] Jimenez Castro PD, Howell SB, Schaefer JJ, Avramenko RW, Gilleard JS and Kaplan RM (2019) Multiple drug resistance in the canine hookworm *Ancylostoma caninum*: An emerging threat? *Parasites and Vectors* 12, 576. 10.1186/s13071-019-3828-6. 31818311 PMC6902405

[ref24] Jimenez Castro PD, Willcox JL, Rochani H, Richmond HL, Martinez HE, Lozoya CE, Savard C and Leutenegger CM (2025) Investigation of risk factors associated with *Ancylostoma* spp. infection and the benzimidazole F167Y resistance marker polymorphism in dogs from the United States. *International Journal of Parasitology Drugs Drug Resist* 27, 100584. 10.1016/j.ijpddr.2025.100584. PMC11847747 39919355

[ref25] Kitchen S, Ratnappan R, Han S, Leasure C, Grill E, Iqbal Z, Granger O, O’Halloran DM and Hawdon JM (2019) Isolation and characterization of a naturally occurring multidrug-resistant strain of the canine hookworm, *Ancylostoma caninum*. *International Journal of Parasitology* 49, 397–406. 10.1016/j.ijpara.2018.12.004. 30771359 PMC6456372

[ref26] Lalosević D, Lalosević V, Klem I, Stanojev-Jovanović D and Pozio E (2008) Pulmonary capillariasis miming bronchial carcinoma. *American Journal of Tropical Medicine and Hygene* 78, 14–16. 18187778

[ref27] Leutenegger CM, Evason MD, Willcox JL, Rochani H, Richmond HL, Meeks C, Lozoya CE, Tereski J, Loo S, Mitchell K, Andrews J and Savard C (2024) Benzimidazole F167Y polymorphism in the canine hookworm, *Ancylostoma caninum*: Widespread geographic, seasonal, age, and breed distribution in United States and Canada dogs. *International Journal for Parasitology: Drugs Drug Resistance* 24, 100520. 10.1016/j.ijpddr.2024.100520. 38237210 PMC10825515

[ref28] Leutenegger CM, Lozoya CE, Tereski J, Andrews J, Mitchell KD, Meeks C, Willcox JL, Freeman G, Richmond HL, Savard C and Evason MD (2023) Comparative study of a broad qPCR panel and centrifugal flotation for detection of gastrointestinal parasites in faecal samples from dogs and cats in the United States. *Parasites and Vectors* 16, 288. 10.1186/s13071-023-05904-z. 37587483 PMC10433665

[ref29] Lopes P, Gomes J, Lozano J, Louro M, De Carvalho LM, Da Fonseca IP, Lobo R, Monteiro F, Carvalho L, Afonso P, Almas M and Cunha MV (2025) Prevalence, diversity and risk factors of gastrointestinal parasites in dogs housed at official shelters across Portugal. *Veterinary Parasitology: Regional Studies and Reports* 62, 101285. 10.1016/j.vprsr.2025.101285. 40518252

[ref30] Márquez-Navarro A, García-Bracamontes G, Álvarez-Fernández BE, Ávila-Caballero LP, Santos-Aranda I, Díaz-Chiguer DL, Sánchez-Manzano RM, Rodríguez-Bataz E and Nogueda-Torres B (2012) *Trichuris vulpis* (Froelich, 1789) Infection in a Child: A Case Report. *The Korean Journal of Parasitology* 50, 69–71. 10.3347/kjp.2012.50.1.69. 22451737 PMC3309054

[ref31] Marsh AE and Lakritz J (2023) Reflecting on the past and fast forwarding to present day anthelmintic resistant *Ancylostoma caninum*–A critical issue we neglected to forecast. *International Journal for Parasitology: Drugs Drug Resistance* 22, 36–43. 10.1016/j.ijpddr.2023.04.003. 37229949 PMC10229760

[ref32] Martin M (2011) Cutadapt removes adapter sequences from high-throughput sequencing reads. *The Global Bioinformatics Network* 17, 10. 10.14806/ej.17.1.200.

[ref33] Merino-Tejedor A, Nejsum P, Mkupasi EM, Johansen MV and Olsen A (2019) Molecular identification of zoonotic hookworm species in dog faeces from Tanzania. *Journal of Helminthology* 93, 313–318. 10.1017/S0022149X18000263. 29606160

[ref34] Mircean V, Cozma V and Gyorke A (2011) *Diagnostic Coproscopic in Bolile Parazitare la Animale, (Coproparasitological Diagnostic in Parasitic Diseases in Animals)*. Cluj-Napoca. Romania: Risoprint, 23–35.

[ref35] Mircean V, Dumitrache MO, Mircean M, Colosi HA and Györke A (2017) Prevalence and risk factors associated with endoparasitic infection in dogs from Transylvania (Romania): A retrospective study. *Veterinary Parasitology* 243, 157–161. 10.1016/j.vetpar.2017.06.028. 28807286

[ref36] Morelli S, Cesare AD, Traversa D, Colombo M, Paoletti B, Ghietti A, Beall M, Davenport K, Buch J, Iorio R, Marchiori E, Di Regalbono AF and Diakou A (2024) Comparison of diagnostic methods for laboratory diagnosis of the zoonotic tapeworm *Dipylidium caninum* in cats. *Veterinary Parasitology* 331, 110274. 10.1016/j.vetpar.2024.110274. 39116546

[ref37] Mukaratirwa S and Singh VP (2010) Prevalence of gastrointestinal parasites of stray dogs impounded by the Society for the Prevention of Cruelty to Animals (SPCA), Durban and Coast, South Africa: Short communication. *Journal of the South African Veterinary Association* 81, 123–125. 10.4102/jsava.v81i2.124. 21247022

[ref38] Niamatali S, Bhopale V and Schad GA (1992) Efficacy of milbemycin oxime against experimentally induced *Ancylostoma caninum* and *Uncinaria stenocephala* infections in dogs. *The Journal of the American Veterinary Medical Association* 201, 1385–1387. 10.2460/javma.1992.201.09.1385. 1429184

[ref39] Ortuno A and Castella J (2011) Intestinal parasites in shelter dogs and risk factors associated with the facility and its management. *Israel Journal of Veterinary Medicine* 66(3).

[ref40] Overgaauw PAM, Vinke CM, Van Hagen MAE and Lipman LJA (2020) A one health perspective on the human–companion animal relationship with emphasis on zoonotic aspects. *International Journal of Environmental Research & Public Health* 17, 3789. 10.3390/ijerph17113789. 32471058 PMC7312520

[ref41] Pal M and Tolawak D (2023) A comprehensive review on major zoonotic parasites from dogs and cats. *International Journal of Medical Parasitology and Epidemiology Sciences* 4, 3–11. 10.34172/ijmpes.2023.02.

[ref42] Papavasili Th A, Kontogeorgos AK, Mavrommati A, Sossidou EN and Chatzitheodoridis F (2024) Review of stray dog management: Dog days in the European countries. *Bulgarian Journal of Veterinary Medicine* 27, 322–342. 10.15547/bjvm.2022-0035.

[ref43] Raza A, Rand J, Qamar AG, Jabbar A and Kopp S (2018) Gastrointestinal parasites in shelter dogs: occurrence, pathology, treatment and risk to shelter workers. *Animals* 8, 108. 10.3390/ani8070108. 30004469 PMC6070783

[ref44] Reinemeyer CR (2016) Formulations and clinical uses of pyrimidine compounds in domestic animals. In *Pyrantel Parasiticide Therapy in Humans and Domestic Animals*. Elsevier, pp. 67–107. 10.1016/B978-0-12-801449-3.00015-6.

[ref45] Rupasinghe R, Chomel BB and Martínez-López B (2022) Climate change and zoonoses: A review of the current status, knowledge gaps, and future trends. *Acta Tropica* 226, 106225. 10.1016/j.actatropica.2021.106225. 34758355

[ref46] Rusu L, Gabriela M, Dumitru A and Liviu M (2023) The prevalence of intestinal parasites in dogs from shelters in Constanța County-Romania. *Scientific Papers - Veterinary Medicine Series* 66, 33–39. 10.61900/SPJVS.2023.04.06.

[ref47] Santana-Hernández KM, Priestnall SL, Modrý D and Rodríguez-Ponce E (2021) Dispersion of adeleid oocysts by vertebrates in Gran Canaria, Spain: Report and literature review. *Parasitology* 148, 1588–1594. 10.1017/S0031182021001244. 35060472 PMC8564802

[ref48] Schwenkenbecher JM, Albonico M, Bickle Q and Kaplan RM (2007) Characterization of beta-tubulin genes in hookworms and investigation of resistance-associated mutations using real-time PCR. *Molecular and Biochemical Parasitology* 156, 167–174. 10.1016/j.molbiopara.2007.07.019. 17850900

[ref49] Sherifi K, Rexhepi A, Hamidi A, Behluli B, Zessin KH, Mathis A and Deplazes P (2011) Detection of patent infections of *Echinococcus granulosus* (“sheep-strain,” G1) in naturally infected dogs in Kosovo. *Berliner Und Munchener Tierarztliche Wochenschrift* 124(11-12), 518–521. 22191174

[ref50] Sommer MF, Zdravković N, Vasić A, Grimm F and Silaghi C (2017) Gastrointestinal parasites in shelter dogs from Belgrade, Serbia. *Veterinary Parasitology: Regional Studies and Reports* 7, 54–57. 10.1016/j.vprsr.2017.01.001. 31014658

[ref51] Soriano SV, Pierangeli NB, Roccia I, Bergagna HFJ, Lazzarini LE, Celescinco A, Saiz MS, Kossman A, Contreras PA, Arias C and Basualdo JA (2010) A wide diversity of zoonotic intestinal parasites infects urban and rural dogs in Neuquén, Patagonia, Argentina. *Veterinary Parasitology* 167, 81–85. 10.1016/j.vetpar.2009.09.048. 19864068

[ref52] Stocker T, Scott I and Šlapeta J (2023) Unambiguous identification of *Ancylostoma caninum* and *Uncinaria stenocephala* in Australian and New Zealand dogs from faecal samples. *Australian Veterinary Journal* 101, 373–376. 10.1111/avj.13272. 37537874

[ref53] Stocker T, Ward MP and Šlapeta J (2024) Nationwide USA re-analysis of amplicon metabarcoding targeting β-tubulin isoform-1 reveals absence of benzimidazole resistant SNPs in Ancylostoma braziliense, *Ancylostoma tubaeforme* and *Uncinaria stenocephala*. *Veterinary Parasitology* 327, 110118. 10.1016/j.vetpar.2024.110118. 38278035

[ref54] Štrkolcová G, Mravcová K, Mucha R, Mulinge E and Schreiberová A (2022) Occurrence of Hookworm and the first molecular and morphometric identification of *Uncinaria stenocephala* in Dogs in Central Europe. *Acta Parasitologica* 67, 764–772. 10.1007/s11686-021-00509-x. 35067865

[ref65] Sulaiman I M, Fayer R, Bern C, Gilman R H, Trout J M, Schantz P M, Das P, Lal A A and Xiao L (2003) Triosephosphate Isomerase Gene Characterization and Potential Zoonotic Transmission of Giardia duodenalis. *Emerging Infectious Diseases* 9(11), 1444–1452. 10.3201/eid0911.030084 14718089 PMC3035538

[ref55] Szwabe K and Blaszkowska J (2017) Stray dogs and cats as potential sources of soil contamination with zoonotic parasites. *Annals of Agricultural and Environmental Medicine* 24, 39–43. 10.5604/12321966.1234003. 28378987

[ref56] Thompson RCA (2023) Zoonotic helminths – Why the challenge remains. *Journal of Helminthology* 97, e21. 10.1017/S0022149X23000020. 36790130

[ref57] Trachsel D, Deplazes P and Mathis A (2007) Identification of taeniid eggs in the faeces from carnivores based on multiplex PCR using targets in mitochondrial DNA. *Parasitology* 134, 911–920. 10.1017/S0031182007002235. 17288631

[ref58] Traversa D (2011) Are we paying too much attention to cardio-pulmonary nematodes and neglecting old-fashioned worms like *Trichuris vulpis*? *Parasites and Vectors* 4, 32. 10.1186/1756-3305-4-32. 21385441 PMC3063211

[ref59] Traversa D, Di Cesare A, Lia RP, Castagna G, Meloni S, Heine J, Strube K, Milillo P, Otranto D, Meckes O and Schaper R (2011) New insights into morphological and biological features of *Capillaria aerophila* (Trichocephalida, Trichuridae). *Parasitology Research* 109, 97–104. 10.1007/s00436-011-2406-4. 21739379

[ref60] Venkatesan A, Jimenez Castro PD, Morosetti A, Horvath H, Chen R, Redman E, Dunn K, Collins JB, Fraser JS, Andersen EC, Kaplan RM and Gilleard JS (2023) Molecular evidence of widespread benzimidazole drug resistance in *Ancylostoma caninum* from domestic dogs throughout the USA and discovery of a novel β-tubulin benzimidazole resistance mutation. *PLOS Pathogens* 19, e1011146. 10.1371/journal.ppat.1011146. 36862759 PMC10013918

[ref61] Von Samson-Himmelstjerna G, Thompson RA, Krücken J, Grant W, Bowman DD, Schnyder M and Deplazes P (2021) Spread of anthelmintic resistance in intestinal helminths of dogs and cats is currently less pronounced than in ruminants and horses – Yet it is of major concern. *International Journal for Parasitology: Drugs Drug Resistance* 17, 36–45. 10.1016/j.ijpddr.2021.07.003. 34343829 PMC8347694

[ref62] Xhekaj B, Stefanovska J, Sherifi K, Rexhepi A, Bizhga B, Rashikj L, Nikolovski M, Kniha E and Cvetkovikj A (2023) Seroprevalence of canine leishmaniosis in asymptomatic dogs in Kosovo. *Parasitology Research* 122, 607–614. 10.1007/s00436-022-07762-7. 36536229

[ref63] Zablan K, Melvin G and Hayley A (2024) Dog ownership, physical activity, loneliness and mental health: A comparison of older adult and younger adult companion animal owners. *BMC Psychology* 12, 618. 10.1186/s40359-024-02104-x. 39487552 PMC11529494

